# Post-procedural *Bacillus cereus* septic arthritis in a patient with systemic lupus erythematosus

**DOI:** 10.4102/ajlm.v9i1.1119

**Published:** 2020-08-20

**Authors:** Barend Mitton, Roxanne Rule, Nontombi Mbelle, Wesley van Hougenhouck-Tulleken, Mohamed Said

**Affiliations:** 1 Department of Medical Microbiology, University of Pretoria, Pretoria, South Africa; 2 Tshwane Academic Division, Department of Medical Microbiology, National Health Laboratory Service, Pretoria, South Africa; 3 Division of Nephrology, Department of Internal Medicine, University of Pretoria, Pretoria, South Africa; 4 Department of Internal Medicine, Steve Biko Academic Hospital, Pretoria, South Africa

**Keywords:** *Bacillus cereus*, septic arthritis, systemic lupus erythematosus, Matrix-assisted laser desorption/ionisation mass spectrometry, MALDI-TOF MS, musculoskeletal infection, arthroscopy

## Abstract

**Introduction:**

*Bacillus* species are often considered as contaminants when cultured from clinical samples. *Bacillus cereus* may be a pathogen in certain circumstances and is known to cause musculoskeletal infections. This report aims to educate clinicians and clinical microbiology laboratories on *B. cereus* musculoskeletal infections and to heighten awareness that *Bacillus* species should not always be dismissed as contaminants.

**Case presentation:**

We report the case of a patient who presented to a tertiary hospital in Pretoria, South Africa, in November 2018 with *B. cereus* septic arthritis and underlying systemic lupus erythematosus (SLE). The isolate would otherwise have been dismissed as a contaminant had it not been for the crucial interaction between the laboratory and the treating clinicians. To our knowledge, this is the first case report of septic arthritis caused by *B. cereus* in an SLE patient where the organism was cultured from the joint specimen. Identification of the organism was performed using matrix-assisted laser desorption/ionisation mass spectrometry.

**Management and outcome:**

Definitive treatment was with intravenous vancomycin, continued for four weeks, in addition to arthroscopy and management of the underlying SLE. The patient had a good clinical outcome and regained full mobility.

**Conclusion:**

Musculoskeletal infections, specifically septic arthritis caused by *B. cereus,* are exceedingly rare infections. Immune suppression, trauma, prosthetic implants and invasive procedures are important risk factors for *B. cereus* musculoskeletal infections. Close collaboration with a multi-disciplinary team approach will effect the best outcome for complicated patients with *B. cereus* infections.

## Introduction

As the vast majority of *Bacillus* species are non-pathogenic and ubiquitous in the environment, many clinical microbiologists and clinicians dismiss *Bacillus* species cultured from clinical specimens as contaminants. Occasionally, this results in a missed diagnosis and inappropriate clinical decision-making. This case is important, because it illustrates the importance of communication between clinicians and the clinical laboratory staff in determining the significance of culture results. This report aims to educate healthcare workers on *Bacillus cereus* joint infections and further endeavours to assist healthcare practitioners in distinguishing when this organism should be dismissed as a contaminant and when it should be considered as a pathogen.

## Ethical considerations

Written, informed consent was obtained from the patient. This research was approved by the University of Pretoria, Faculty of Health Sciences, Research Ethics Committee (ethics reference number 133/2019).

## Case presentation

In November 2018, a 32-year-old male was referred to a tertiary academic hospital in Pretoria, South Africa, with a non-resolving septic arthritis of his right knee. The patient presented to a secondary hospital 10 days prior with a tender, swollen right knee, with no history of trauma. He underwent an arthroscopy at that centre and received intravenous amoxicillin-clavulanic acid, with a suboptimal response. In addition, he developed symptoms suggestive of systemic lupus erythematosus (SLE), which included polyarthritis, xerostomia, Raynaud’s phenomenon, proteinuria and confusion. On examination he was haemodynamically stable with a pulse rate of 100 beats per minute, a blood pressure of 117/81 mmHg and a temperature of 36.5 °C. He had asymmetric polyarthritis, involving the right elbow, left wrist, right knee and both ankles. The right knee was the worst affected, with swelling, erythema and tenderness on examination. An emergency arthroscopy of the right knee was performed, revealing a purulent effusion. Intravenous ceftriaxone (1 g twice daily) was started empirically. X-rays of all affected joints revealed no accompanying osteomyelitis. No other imaging of the joints was done. The diagnosis of SLE was confirmed based on a Systemic Lupus International Collaborating Clinics score^1^ of five (anti-nuclear antibody positive, lupus nephritis class 3, arthritis, low C3 and neurologic SLE).

### Laboratory investigations

**FIGURE 1:**
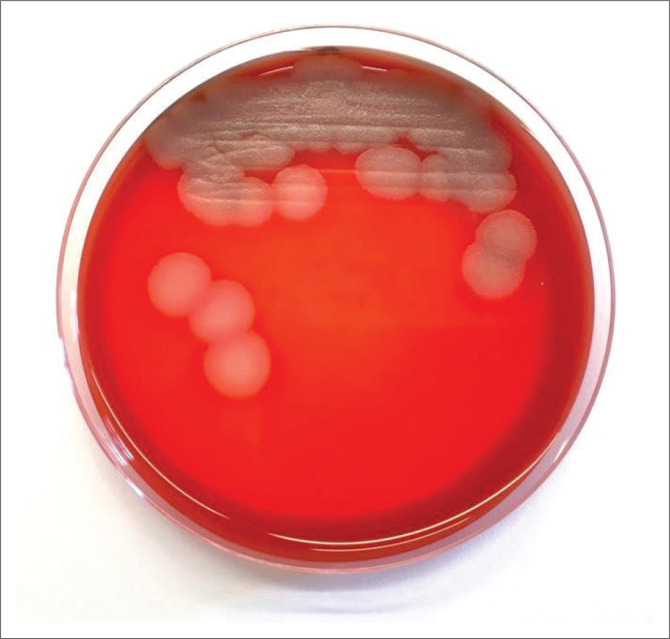
Typical morphology of *Bacillus cereus* on 5% horse blood agar (Pretoria, South Africa, 19 March 2019).

Admission blood test revealed a white cell count of 8.04 × 10^9^ cells/L with neutrophilia (73%), a C-reactive protein of 107 mg/L, a positive anti-nuclear antibody (titre 160) and a low C3 (0.50 g/L). In addition, an Epstein–Barr virus viraemia of 530 copies/mL was found. Admission blood cultures had no growth. A pus sample taken during arthroscopy showed numerous Gram-positive bacilli on the direct Gram stain. Culture revealed large, flat, grey, beta-haemolytic colonies on 5% horse blood agar (Figure 1), which also grew on chocolate agar and MacConkey agar. This isolate was identified as *Bacillus* species and reported as a possible contaminant. The patient had a poor clinical response to the empiric ceftriaxone after 6 days of treatment, and a request was made by the attending clinicians for antimicrobial susceptibility testing on the isolate. The isolate was therefore referred for further identification to species level using matrix-assisted laser desorption/ionisation mass spectrometry by means of VITEK^®^ MS (bioMérieux, Marcy l’Etoile, France), instrument software version 1.5.0.4, MYLA^®^ version 4.5.1 (bioMérieux), Knowledge Base (database) version 3.2. The isolate was identified as *Bacillus cereus*. Antibiotic susceptibilities were performed by ETEST^®^ (bioMérieux, Marcy l’Etoile, France) and interpreted using the Clinical & Laboratory Standards Institute M45 (2015) breakpoints.^2^ The isolate was resistant to penicillin (minimum inhibitory concentration [MIC] 32 *µ*g/mL), and cefotaxime (MIC 32 *µ*g/mL), but susceptible to vancomycin (MIC 4 *µ*g/mL) and imipenem (MIC 4 *µ*g/mL).

## Management and outcome

Based on the report, the patient was started on intravenous vancomycin 1 g twice daily. The dose was adjusted to achieve a target vancomycin blood trough level of 15 mg/mL – 20 mg/mL. Trough levels were monitored roughly every 3 days over the treatment period, and the dosage adjusted accordingly. No vancomycin-related adverse events occurred during this time. Vancomycin was continued for 4 weeks; over this period, the pain and swelling improved dramatically, inflammatory markers normalised and the patient regained mobility. Management of the SLE included prednisone, mycophenolate mofetil and chloroquine, with good response.

## Discussion

*Bacillus* species are Gram-positive, aerobic or facultative anaerobic sporulating bacilli, which are ubiquitous in the environment.^3^ Over 100 species are known to belong to the genus.^3^ *Bacillus cereus* is a common cause of food poisoning and occasionally causes opportunistic infections, usually in vulnerable hosts. These infections include ophthalmic infections, wound infections, septicaemia, endocarditis, meningitis, necrotising pneumonia and orthopaedic infections.^3,4^ Important virulence factors of *B. cereus* include production of toxins and the formation of biofilms and spores.^4^ Except for *B. cereus* and *B. anthracis*, the genus *Bacillus* is rarely associated with disease.^3,5,6,7^ Therefore, *Bacillus* species are often reported as contaminants, even when cultured from sterile specimens. This may result in a delay in correct diagnosis and inappropriate treatment.

**FIGURE 2:**
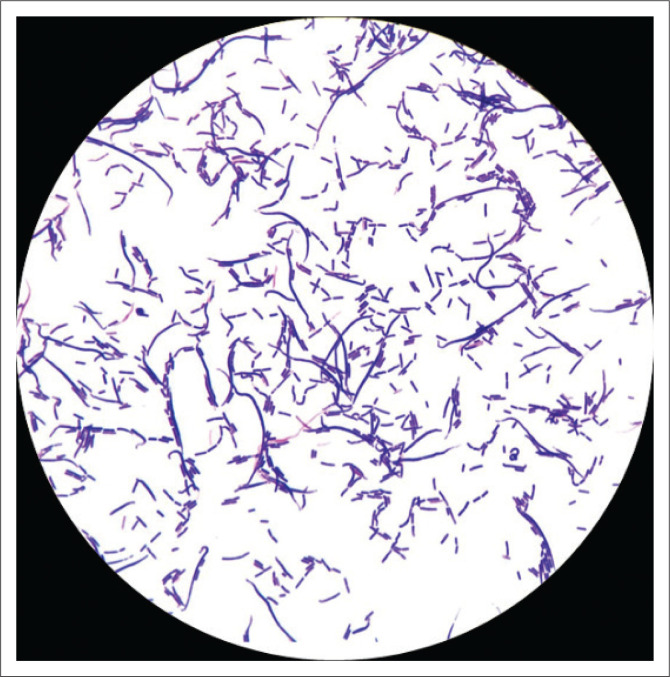
Microscopic image at 1000 × magnification showing boxcar-shaped, Gram-positive bacilli, typical of *Bacillus cereus* (Pretoria, South Africa, 19 March 2019).

*Bacillus cereus* grows well on most routine culture media such as blood agar (Figure 1), chocolate agar and MacConkey agar. Routine laboratory tests will reveal large boxcar-shaped, Gram-positive bacilli (Figure 2) that are catalase positive. However, identification to species level requires specialised techniques such as matrix-assisted laser desorption/ionisation mass spectrometry or molecular techniques.^7^

Musculoskeletal infections caused by *B. cereus* are rare but have been previously reported in literature.^4,5,6,7^ Åkesson, Hedströum and Ripa.^4^ reported 12 cases of *B. cereus* orthopaedic infections, all occurring in post-operative or post-traumatic wounds, whilst Dubouix et al.^5^ reported 41 cases with *B. cereus* wound infections associated with open fractures. Gallo et al.^6^ reported two cases of *B. cereus* prosthesis-related septic arthritis, which were culture negative but identified using PCR-mass-spectrometric-technology and fluorescence in situ hybridisation of tissue. Ha et al.^7^ reported a single case of late prosthetic joint infection with *B. cereus* that occurred 13 years after total hip replacement surgery, confirmed by 16S ribosomal ribonucleic acid (rRNA) sequencing. In total, we found 56 cases reported over 25 years, highlighting the rarity of *B. cereus* musculoskeletal infections and emphasising the difficulty in making a definitive diagnosis. The scarcity of these infections may actually be the result of under-reporting, as *Bacillus* species are often reported as contaminants.

*Bacillus cereus* produces β-lactamases and is resistant to most β-lactam antibiotics, except carbapenems.^3^ Classically, *Bacillus* species are susceptible to vancomycin, aminoglycosides and fluoroquinolones, whilst resistance to erythromycin, tetracycline and even carbapenems have been reported.^3^ The empiric antibiotic of choice for invasive *B. cereus* infections is vancomycin.^3,7^ In addition to administering antibiotics, it is imperative to obtain source control at the infected site, as *B. cereus* is known to form biofilm. The removal of implanted medical devices may be necessary.^7^

The concomitant SLE may be considered a notable risk factor for invasive *B. cereus* infection in this patient. It is uncertain if the Epstein–Barr virus viraemia played a significant role in predisposing the patient further to this infection. However, both SLE and Epstein–Barr virus infection are known to be immune–modulatory, down-regulating the innate and humoral systems and placing the patient at increased risk of opportunistic infections.^8,9^ An additional risk factor was the preceding arthroscopy, which may have introduced spores into the joint space. To our knowledge, this is the first case of septic arthritis in an SLE patient where the organism was cultured from the joint specimen. The communication between the clinical and microbiology teams ensured that the organism was identified to species level and that antibiotic susceptibility testing was performed, resulting in a favourable outcome for the patient.

### Conclusion

*Bacillus* species are often regarded as contaminants and receive little attention from the medical community. In certain high-risk patient groups, however, *B. cereus* may be a formidable pathogen. Clinical microbiology laboratorians and clinicians should have a high index of suspicion in these patients and identify *Bacillus* species in cultures from sterile sites to species level as well as perform antibiotic susceptibility when *B. cereus* is identified. Immune suppression, trauma, prosthetic implants and invasive procedures are important risk factors for *B. cereus* musculoskeletal infections. *Bacillus cereus* is universally resistant to most β-lactam antibiotics, with the exception of carbapenems. Treatment with vancomycin was successful in the case described. Close collaboration with a multi-disciplinary team approach will effect the best outcome for complicated patients with *B. cereus* infections.
